# Accelerating progress towards the elimination of mother‐to‐child transmission of HIV: a narrative review

**DOI:** 10.1002/jia2.25571

**Published:** 2020-08-20

**Authors:** Benjamin H Chi, Dorothy Mbori‐Ngacha, Shaffiq Essajee, Lynne M Mofenson, Fatima Tsiouris, Mary Mahy, Chewe Luo

**Affiliations:** ^1^ University of North Carolina at Chapel Hill Chapel Hill NC USA; ^2^ United Nations Children’s Fund (UNICEF) New York NY USA; ^3^ Elizabeth Glaser Pediatric AIDS Foundation Washington DC USA; ^4^ ICAP at Columbia University New York NY USA; ^5^ Joint United Nations Programme on HIV/AIDS (UNAIDS) Geneva Switzerland

**Keywords:** HIV prevention, children, elimination of mother‐to‐child transmission, prevention of mother‐to‐child transmission, global

## Abstract

**Introduction:**

Findings from biomedical, behavioural and implementation studies provide a rich foundation to guide programmatic efforts for the prevention of mother‐to‐child HIV transmission (PMTCT).

**Methods:**

We summarized the current evidence base to support policy makers, programme managers, funding agencies and other stakeholders in designing and optimizing PMTCT programmes. We searched the scientific literature for PMTCT interventions in the era of universal antiretroviral therapy for pregnant and breastfeeding women (i.e. 2013 onward). Where evidence was sparse, relevant studies from the general HIV treatment literature or from prior eras of PMTCT programme implementation were also considered. Studies were organized into six categories: HIV prevention services for women, timely access to HIV testing, timely access to ART, programme retention and adherence support, timely engagement in antenatal care and services for infants at highest risk of HIV acquisition. These were mapped to specific missed opportunities identified by the UNAIDS Spectrum model and embedded in UNICEF operational guidance to optimize PMTCT services.

**Results and discussion:**

From May to November 2019, we identified numerous promising, evidence‐based strategies that, properly tailored and adopted, could contribute to population reductions in vertical HIV transmission. These spanned the HIV and maternal and child health literature, emphasizing the importance of continued alignment and integration of services. We observed overlap between several intervention domains, suggesting potential for synergies and increased downstream impact. Common themes included integration of facility‐based healthcare; decentralization of health services from facilities to communities; and engagement of partners, peers and lay workers for social support. Approaches to ensure early HIV diagnosis and treatment prior to pregnancy would strengthen care across the maternal lifespan and should be promoted in the context of PMTCT.

**Conclusions:**

A wide range of effective strategies exist to improve PMTCT access, uptake and retention. Programmes should carefully consider, prioritize and plan those that are most appropriate for the local setting and best address existing gaps in PMTCT health services.

## 1 INTRODUCTION

Significant achievements have been made in the prevention of mother‐to‐child HIV transmission (PMTCT), transforming the paediatric HIV epidemic globally. With new innovations, strong political will and rapid programme expansion, the number of new child infections resulting from vertical transmission has decreased dramatically in nearly two decades – from over 400,000 in 2000 to 160,000 in 2018 [1]. Despite these substantial gains, however, the pace towards reaching the global goals for ending AIDS has slowed. The estimated 160,000 new HIV infections in children globally in 2018 was four times the target of 40,000 set forth by the *Start Free Stay Free AIDS Free* initiative [1]. At the current trajectory, the target for the year 2020 (fewer than 20,000 new child HIV infections) – and the goals for the elimination of mother‐to‐child HIV transmission (EMTCT) – are in jeopardy.

To help address this challenge, the United Nations Children’s Fund (UNICEF) and partners released *Going the ‘Last Mile’ to EMTCT: A roadmap for ending the HIV epidemic in children* in February 2020 [2]. This document (shortened to the *Last Mile to EMTCT* in this article) describes a data‐driven approach to iteratively assess, plan and implement PMTCT interventions tailored to local needs and priorities. Four steps are outlined, comprising eight distinct activities, to provide a framework for planned action and implementation (Table 1). First, to ensure a collaborative approach with multiple stakeholders, country teams are formed to guide the deliberative process. Second, country‐developed estimates from the UNAIDS Spectrum model, triangulated with additional locally available data, are used to identify the missed opportunities for preventing new child HIV infections in the country and the programmatic gaps that may contribute to them. Third, country teams prioritize and plan strategies that may best address these identified gaps, drawing from programmatic experiences and evidence‐based practices. Finally, plans are disseminated, monitored and evaluated to ensure that they deliver on their intended promise.

**Table 1:** The structured steps and activities for the *Last Mile to EMTCT*. Table is adapted from [2] and published with permission from UNICEF

Step 1. Developing a consultative process	
*Activity 1:* Identify a country team to drive assessment and planning processes	A team approach, one that represents the diverse perspectives of key stakeholders, is critical to the success of this planning process. Team members should be identified at the start of the process and include representatives from local government (including ministries of health), national AIDS organizations, national HIV estimates teams, UN agencies, implementing partners, funding agencies, academicians and researchers, and community stakeholders. Where possible, this should be built upon existing government structures, including technical working groups, EMTCT national validation committees and other existing groups
Step 2. Taking stock of progress and remaining gaps in PMTCT	
*Activity 2:* Conduct a missed opportunity analysis	We recommend use of the UNAIDS Spectrum to identify missed opportunities at the national and (where possible) subnational levels. The Spectrum stacked bar can provide proportional estimates of the causes of new child HIV infections in a given country or region
*Activity 3:* Characterize and contextualize programmatic gaps using data from available sources	While the missed opportunity analysis identifies groups in need of PMTCT services, data from other sources are used to characterize and contextualize the programmatic gaps. This information can provide a clearer picture of where and when these new infant HIV infections occur
Step 3. Planning and prioritizing	
*Activity 4:* Articulate the priority factors that are necessary for programmatic change	PMTCT services should be tailored to the local context. This should be a participatory process – including members of the country team – to identify those intervention characteristics that should be considered for widespread and effective implementation
*Activity 5:* Prioritize interventions according to gaps and contextual factors	Country teams review relevant and resource‐appropriate interventions and strategies to address identified programmatic gaps and reduce the number of new infant HIV infections. These are then prioritized according to the key contextual factors articulated in Activity 4
*Activity 6:* Seek broader stakeholder engagement and finalize strategies, guidelines and/or policies	Once a set of strategies, guidelines and/or policies have been agreed upon, it should be vetted more broadly across different stakeholder groups. This input can help the country team to further refine their proposed changes, with particular focus on implementation
Step 4. Implementing, monitoring and evaluating for PMTCT	
*Activity 7:* Disseminate planned strategies, guidelines and/or policies	For most countries, dissemination procedures are established at the national level. Once finalized, planned PMTCT activities should be disseminated according to those practices. Accompanying materials for implementation guidance, monitoring and evaluation, and community outreach should be developed and disseminated
*Activity 8:* Monitor and evaluate implemented interventions	The successful implementation of new policies typically requires ongoing monitoring and evaluation. Such practices should be designed early and implemented alongside the PMTCT interventions themselves. Data reports and real‐time dashboards can be used to drive programmatic change and serve as the foundation for quality improvement efforts at the provincial, district and facility levels

The third of these four steps – where programmes plan and prioritize effective strategies to address the prevailing gaps in local PMTCT services – is crucial. To inform this process, we reviewed the scientific literature for promising and evidence‐based interventions that may help programmes meet the validation criteria for EMTCT at the national level, including the path to elimination milestones.

## 2 METHODS

**Figure 1:**
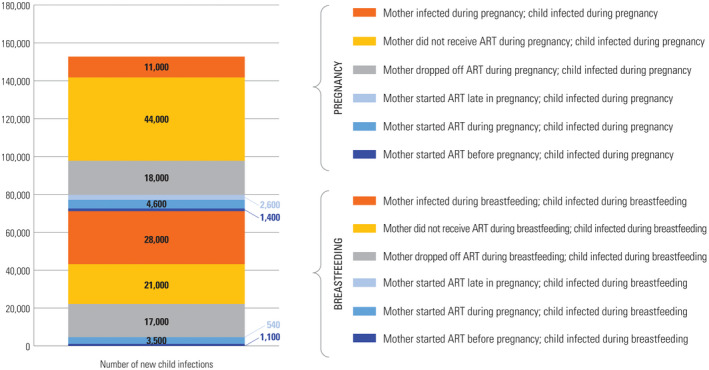
The global “stacked bar” analysis generated from the UNAIDS Spectrum model, showing the estimated number of total new child HIV infections worldwide in 2018 and their attributable causes. Figure is adapted from [2] and published with permission from UNICEF.

**Figure 2:**
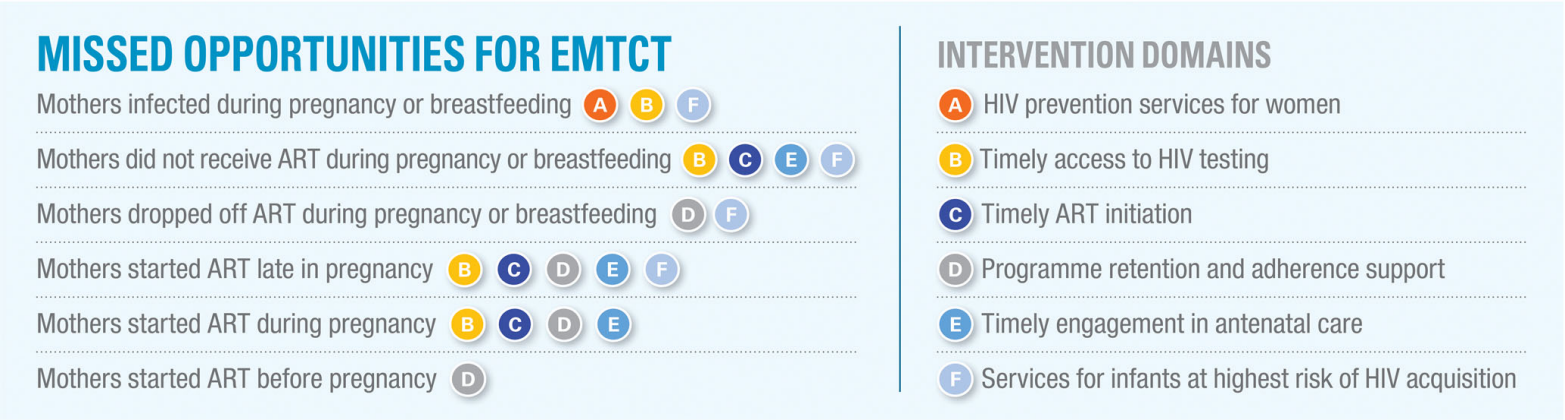
The missed opportunities for EMTCT, as determined by the Spectrum stacked bar analysis, mapped to specific intervention domains. Figure is adapted from [2] and published with permission from UNICEF.

The Spectrum PMTCT “stacked bar” analysis (Figure 1) uses programmatic and modelled data to estimate new child HIV infections and attributes those infections to one of six groups: mothers who newly acquired HIV, mothers who received no antiretroviral prophylaxis or treatment, mothers who previously started ART but stopped, mothers who started ART just before delivery, mothers who started ART during pregnancy and mothers who started ART before pregnancy [1]. Children living with HIV are further stratified by whether they acquired HIV during pregnancy or breastfeeding. We identified six intervention domains, mapped to these missed opportunity groups described by the Spectrum PMTCT stacked bar (Figure 2): HIV prevention services for women, timely access to HIV testing, timely access to ART, programme retention and adherence support, timely engagement in antenatal care and services for infants at highest risk of HIV acquisition. These domains provide the underlying framework for our literature review.

From May to November 2019, we conducted a narrative review to identify interventions that could be tailored to address identified gaps and needs in PMTCT programming. We focused on studies of pregnant and/or breastfeeding women conducted from 2013 onward, a period aligned with the World Health Organization (WHO)’s adoption of universal antiretroviral therapy (ART) in these populations [3]. We included biomedical, behavioural and implementation strategies; no exclusions were made in terms of design. We searched PubMed to identify relevant publications in the medical and public health literature. All studies were categorized by their relevance to the six above‐mentioned intervention domains. Where evidence was sparse, relevant studies from the general HIV treatment literature or from prior eras of PMTCT programme implementation were also considered. Our literature search was further enriched via two approaches. Bibliographies from selected articles were reviewed to further identify sources in peer‐reviewed journals, conference proceedings, grey literature and international clinical and policy guidelines. We also relied on expert opinion – including the advisory committee for the *Last Mile to EMTCT* guidance – for additional sources. These studies were critically reviewed and evaluated prior to their inclusion in this narrative review.

## 3 RESULTS

### 3.1 HIV prevention services for women

A growing body of work confirms the high incidence of HIV among pregnant and breastfeeding women in many sub‐Saharan Africa settings [4, 5]. New HIV infections have long‐term consequences to the individual, including increased lifetime morbidity and mortality. During pregnancy and breastfeeding, however, there are additional concerns around mother‐to‐child HIV transmission. Women who are newly infected with HIV during pregnancy have an 18% chance of transmitting HIV to her newborn; when the incident maternal HIV infection occurs during breastfeeding, this probability may be as high as 27% [6]. Even in settings of high HIV prevalence and high ART coverage, new but undiagnosed HIV infection in pregnant and breastfeeding women may account for a growing proportion of new infant HIV infections [7], as high as 43% in some settings [8].

A couples‐based framework may be useful to guide HIV prevention during pregnancy and breastfeeding [9]. When pregnant women know their HIV status and that of their partners, they can better understand their individual risks for HIV acquisition/transmission and access appropriate HIV prevention, care and treatment services. According to this model, a vital early step is HIV testing for both the index pregnant woman and her partner(s). While HIV testing services have expanded rapidly in antenatal settings, programmatic efforts to engage male partners have not kept pace and, even where attempted, have faced limited success [10]. Many PMTCT programmes offer facility‐based couples counselling and these services can be enhanced with different options for partner notification [11]. In randomized trials in Kenya, home‐based HIV testing for couples was shown to be acceptable, feasible and cost‐effective [12, 13, 14]. Provision of HIV self‐test kits may provide another important avenue for HIV testing that is further decentralized. Women are taught how to administer an HIV self‐test and then sent home with HIV self‐test kits for her and/or her partner [15, 16]. Given the high efficacy of ART in reducing horizontal HIV transmission [17, 18], rapid ART initiation following HIV diagnosis of male partners of HIV‐negative pregnant women is paramount. Programmes that promote couples‐ or family based ART delivery models – including those integrated within maternal and child health services – may increase the likelihood of early uptake, initiation and adherence of HIV treatment.

Despite high burden of new HIV infections during pregnancy and breastfeeding, women initially testing HIV‐negative during antenatal care often do not receive HIV prevention services following post‐test counselling. A number of behavioural interventions have been proposed, but results thus far have been mixed. Jones and colleagues, for example studied a combination intervention comprising two evidence‐based components: a couples’ behavioural risk reduction and an intervention designed to enhance PMTCT uptake. The intervention was associated with decreases in unprotected sex and increased HIV knowledge; while the number of new maternal HIV infections was small, none occurred among women in the intervention arm (vs. six in the control arm) [19]. Homsy and colleagues found that an enhanced, longitudinal HIV counselling and testing strategy designed to prevent HIV acquisition in pregnant women did not result in significant differences in behavioural outcomes such as reported condom use. No differences were noted in HIV incidence, but observed rates were low overall (0.2 per 100 person‐years) [20]. When HIV‐negative women were provided community health worker support in South Africa – including individualized HIV counselling, regular individual‐ and couples‐based HIV testing and referral services for male partners (e.g. circumcision, treatment of sexually transmitted infections, HIV treatment) – low rates of incident HIV infection were observed antenatally (1.49 per 100 women‐years) and postnatally (1.03 per 100 women‐years) [21]. Although direct comparisons to care without the community health worker component were not made as part of the study, rates were substantially lower than that of prior studies in the region [4]. Given varying results of such interventions to date, it would be critical to include an evaluation component if piloting or implementing such prevention strategies.

PrEP has an important role in HIV prevention, including for pregnant and breastfeeding women [22, 23, 24, 25]. When adherence is maintained, PrEP – formulated as once daily tenofovir disoproxil fumarate and emtricitabine (TDF‐FTC) – has been shown to be highly effective across numerous randomized trials in women [26]. TDF‐FTC has been shown to be safe in studies of HIV‐positive pregnant women receiving TDF‐based ART, hepatitis‐B mono‐infected pregnant women receiving TDF alone, and HIV‐negative women receiving PrEP at the time they became pregnant or while they were pregnant [27, 28, 29, 30, 31, 32]. Although the WHO supports its use during pregnancy and breastfeeding [33], many national programs have not adopted full‐scale implementation for pregnant and breastfeeding women [34]. Nevertheless, early studies support the promise of PrEP in these populations. In an evaluation of PrEP delivery in 16 maternal and child health clinics in Kenya, 22% initiated PrEP. Pregnant women with known HIV‐positive partners most frequently initiated PrEP (79%) and this was an important predictor of PrEP continuation [35]. Acceptability and feasibility have been reported in qualitative studies from the region [36, 37, 38, 39]. Modelling studies also suggest that integration of PrEP into antenatal services could significantly reduce the number of new HIV infections [40, 41]. The identification of pregnant and breastfeeding women at highest risk of HIV acquisition could make this potential yield even greater. In Kenya, Pintye and colleagues developed a risk score to identify and prioritize HIV‐negative pregnant and breastfeeding women to HIV pre‐exposure prophylaxis (PrEP) use, one that included lifetime sexual partners, male partner HIV status and syphilis status [42]. A cluster‐randomized trial is currently underway to compare the population impact of HIV risk screening versus universal access approaches to PrEP during pregnancy [43].

While primary HIV prevention is an important pillar of PMTCT, some women may acquire HIV during pregnancy and breastfeeding despite the best programmatic efforts. Strategies are needed to diagnose such women as early as possible, so that PMTCT interventions – including ART – may be rapidly initiated. The WHO currently recommends repeat HIV testing for pregnant women with initial HIV negative test results, beginning in the third trimester and continuing postpartum during breastfeeding [44]. In a recent review of 49 national HIV testing policies, 78% recommended repeat HIV testing. Eleven national guidelines did not include HIV retesting, including several countries identified as priority countries for PMTCT because of their high burden of HIV [45]. In settings where repeat HIV testing is prioritized, programmes should focus on missed opportunities for retesting (e.g. poor retention in antenatal care [46]); integration of testing into routine health platforms, including maternal and child health services; and use of newer HIV testing modalities (e.g. HIV self‐testing, home‐based testing).

### 3.2 Timely access to HIV testing

Diagnosis of HIV is essential to enter the PMTCT continuum of care. The earlier this can be accomplished, the greater the likelihood of virological suppression prior to delivery; this in turn dramatically reduces the risk for mother‐to‐child transmission of HIV. Based on a synthesis of the existing literature, for example the UNAIDS Spectrum model estimates a 0.3% risk of peripartum HIV transmission if a woman with HIV initiated ART prior to conception, a 1.4% risk when initiated at 14 weeks gestation, and an 8.2% risk when initiated less than four weeks from delivery [47]. For women who test HIV‐negative, post‐test counselling can provide opportunities to discuss risk reduction and repeat HIV testing over the course of pregnancy and breastfeeding. Additionally, it may empower women to request recommended HIV prevention services that might otherwise be missed.

Widespread availability of rapid HIV antibody tests – assays that are high performing, inexpensive, easy to store, and can provide results quickly – has greatly increased access to these services. In many settings, there has been widespread adoption of “opt‐out” HIV testing during pregnancy [48], a strategy that decreases stigma by incorporating it into routine antenatal services. Integration of HIV counselling and testing into already busy antenatal clinics has required task‐shifting, including use of trained lay counsellors [49, 50]. HIV testing should be performed early, ideally at a pregnant women’s initial enrolment into antenatal care. Repeat HIV testing in the third trimester, during labour, and/or during breastfeeding can further identify women who are newly infected with HIV, so they may enrol in long‐term HIV care and initiate ART. Studies have shown that the addition of a second HIV test during pregnancy for women initially testing HIV‐negative may be cost‐effective across different settings [51, 52].

Strategies that decentralize HIV testing – either outside of the health facility or out of the hands of overburdened facility‐based providers – have been shown to be effective. Ezeanolue and colleagues, for example demonstrated the effectiveness of a congregation‐based strategy to increase HIV testing among pregnant women in a cluster‐randomized trial across 40 churches in Nigeria. When HIV testing was integrated into church‐led “baby showers” (n = 3002), the rates of HIV testing increased significantly compared to those receiving standard referrals (92% vs. 55%) [53]. Integration of HIV testing into maternal, newborn and child health (MNCH) “weeks” (biannual campaign‐style events designed to expand health service access) may also be promising. In a single week, across 13 local government areas in Nigeria’s Benue State, more than 50,000 pregnant women were educated about HIV testing and >99% subsequently underwent testing via an *opt out* approach [54]. Risk stratification and other triage strategies should be used to increase the efficiency of such testing approaches in identifying pregnant women living with HIV. Such services should also complement strong existing HIV programmes, to ensure linkages to follow‐on health services.

### 3.3 Timely access to ART

Once women are diagnosed with HIV, ART initiation should commence as soon as possible. Geographic coverage of HIV care and treatment should be critically evaluated, to ensure that HIV‐positive women have ready access to these services. Distance to health facilities has been inversely associated with service uptake [55, 56] and this should be considered in planning. Other structural barriers, including user fees and waiting times, should be examined and addressed where possible.

Even when services are available at nearby facilities – or even in different units at the same health facility – additional support may be needed. Studies investigating the integration of HIV and maternal and child health services have been encouraging. In a cluster‐randomized study in rural Kenya, for example Turan and colleagues showed that integrating antenatal care and HIV care led to greater HIV care enrolment (69% vs. 36%; odds ratio: 3.94, 95% CI: 1.14 to 13.63) and higher ART initiation (40% vs. 17%, odds ratio: 3.22, 95% CI: 1.81 to 5.72) compared to the standard of care [57]. Similar results were reported from a stepped wedge evaluation in Zambia, where integrated services were associated with increases in HIV care enrolment (44.4% vs. 25.3%, adjusted odds ratio: 2.06, 95% CI: 1.27 to 3.34) and ART initiation (32.9% vs. 14.4%, adjusted odds ratio: 2.01, 95% CI: 1.37 to 2.95) [58]. In a retrospective analysis in Malawi, full integration of HIV testing and ART provision within antenatal care resulted in significantly higher ART initiation than partial integration model (HIV testing only, referrals for ART initiation; 63% vs. 51%). However, the fully integrated model was also associated with lower retention (79% vs. 87%) [59]. Given the importance of rapid ART initiation, additional support should be given to new starters so the risk for early default is minimized. Other studies of integrated HIV and maternal‐child healthcare have largely echoed these study findings, highlighting the importance of such integrated care models over the course of pregnancy and breastfeeding to promote adherence and programme retention [60, 61, 62, 63, 64].

### 3.4 Programme retention and adherence support

To reach the ambitious targets of EMTCT, ART adherence and programme retention are critical; unfortunately, both present unique challenges over the course of pregnancy and breastfeeding [65, 66, 67, 68]. In their review of the literature, Stover, et al. estimated that maternal PMTCT retention to ART programmes at 80% at time of delivery [47]. Different forms of peer support have been shown to be effective [69]. The “mentor mothers” approach, for example trains HIV‐positive women with PMTCT experience to provide education, psychosocial support and operational guidance navigating the health system. In a comparative cohort study in Nigeria, women receiving mentor mother support had significantly higher odds of retention (adjusted odds ratio: 5.9, 95% CI: 3.0 to 11.6) and viral suppression (adjusted odds ratio: 4.9, 95% CI: 2.6 to 9.2) at six months postpartum [70]. The PURE Malawi trial found that, compared to the prevailing standard of care, facility‐ and community‐based peer support models led to better uptake of ART (81% vs. 86% and 90% respectively) and retention at 24 months (66% vs. 80% and 83% respectively) [71]. Other studies have shown that peer‐centred approaches can effectively be combined with other interventions [72] and encourage other reproductive health behaviours [73]. These trial findings are largely supported by qualitative research, which confirm the overall feasibility and acceptability of the intervention [74, 75, 76], including among adolescents [77]. For widespread implementation, however, adaptations may be required to fully optimize the role of peer supporters within the health system [78].

Community‐level health providers may also serve in this supporting role. The MIR4Health study, for example deployed trained lay counsellors to provide pregnant women with coordinated support, including individualized health education, retention/adherence support, phone and SMS appointment reminders and missed visit tracking. Women in the intervention arm had lower rates of attrition by six months postpartum when compared to the standard of care (18.8% vs. 28.2%, relative risk: 0.67, 95% CI: 0.45 to 0.99) [79].

Identifying support within the woman’s own social network may also increase adherence and retention. Engagement of male partners has been shown to improve progression along the PMTCT cascade, including uptake of HIV testing and ART among HIV‐positive women [80, 81]. Partner involvement has also been shown to improve outcomes among HIV‐exposed infants, including new HIV infections and overall HIV‐free survival [82, 83, 84]. Conversely, a lack of engagement can have negative consequences. In a cross‐sectional study in Malawi, couples in which neither partner disclosed their HIV status was associated with higher risk of not initiating maternal ART (adjusted odds ratio: 4.7, 95% CI: 2.5 to 8.8), suboptimal treatment adherence (adjusted odds ratio: 1.8, 95% CI: 1.1 to 2.8) and HIV transmission from mother to infant (adjusted odds ratio: 2.1, 95% CI: 1.1 to 4.1) [85]. Strategies to increase male partner engagement have typically started with couples HIV counselling and testing; community‐based outreach activities to engage male partners have also been effective in Mozambique and Tanzania [80, 86]. However, the potential risks associated with these approaches, including intimate partner violence and social harms, should be carefully considered when implementing such services at a population level.

Other evidence‐based strategies, shown to be effective in different populations, may be adapted for pregnant and breastfeeding women. Community‐ or facility‐based adherence groups, for example, have shown promise among “stable” ART patients who have already achieved virological suppression. Healthcare staff convene patients – typically every few months – for group counselling, prescription refills and referrals to the health facility (where needed). In a cohort study of 129 women with HIV viral load <1000 copies/mL for at least three months, Myer and colleagues reported favourable outcomes with adherence groups during the postpartum period [87]. The feasibility and acceptability of the approach was confirmed via in‐depth interviews with healthcare providers and patients [88]. Although encouraging, further research is needed about patient preferences and long‐term outcomes in this population. Similarly, mobile health (or mHealth) technologies have been shown to improve retention and adherence across numerous studies in the general adult HIV population but studies focused on antenatal and postpartum populations have been limited. In separate trials, two‐way messaging and biweekly phone calls were associated with increased retention within the first eight to ten weeks following delivery [88, 89]; however, data for longer term retention (i.e. out to 12 months postpartum) appear equivocal [90]. Such approaches deserve further study, especially given the growing landscape for mHealth in maternal and child services [91], but their population‐level effectiveness will likely depend on evolving structural, social and cultural factors.

Several strategies appear promising for pregnant and breastfeeding women but require further evaluation. Although viral load monitoring is now recommended in many settings, coverage remains limited and its timing infrequent (e.g. six to twenty‐four months apart). Without intensified monitoring, as many as 70% may not receive routine viral load testing during pregnancy and breastfeeding, with potentially negative consequences [92]. Viral load testing at entry into antenatal care (for women on ART prior to conception) can help to identify undiagnosed treatment failure. Similar testing in the third trimester and/or during breastfeeding can used to guide adherence counselling and support, while identifying the need for enhanced neonatal antiretroviral prophylaxis. Due to the delays in turnaround time, particularly in remote and rural areas, the integration of point‐of‐care (POC) viral load assays can increase coverage of virological monitoring. However, such POC instruments should be placed in a way that maximizes centralized laboratories (and their existing specimen transport networks) and minimizes overall costs [93, 94].

Rapid viral load reductions can be achieved with integrase‐inhibitor‐based ART regimens, which may be particularly important for women first presenting late in pregnancy. In pregnant women with detectable viral load on an existing non‐integrase inhibitor regimen, switching to an integrase inhibitor‐based ART regimen may provide rapid viral load reduction [95]; however, in this situation, a change in the nucleoside reverse transcriptase (NRTI) backbone may also be needed to ensure two active drugs are being used when making a switch [96]. Despite initial concerns about pre‐conception dolutegravir use and its association with foetal neural tube defects [97], subsequent data suggest that the overall risks were small (an observed 0.30% neural tube defect prevalence with pre‐conception dolutegravir) and there was no difference in other adverse birth outcomes compared to pre‐conception efavirenz regimens [98]. Initiation of dolutegravir‐containing regimens during pregnancy was not associated with elevated risk for adverse birth outcomes, when compared to efavirenz‐containing regimens [99]. This led the WHO to recommend dolutegravir‐based regimens as part of first‐line ART for all populations, including pregnant women [96].

### 3.5 Timely engagement in antenatal care

Early and continued engagement in antenatal care is foundational to strong PMTCT programmes. Delayed registration for antenatal care may diminish the time for HIV testing and ART initiation among pregnant women not yet aware of their HIV status. While the WHO has expanded the minimum number of recommended contacts from four to eight [100], novel approaches are needed to promote antenatal care engagement as early as the first trimester.

To date, evidence for community‐based interventions to support antenatal care engagement has been mixed. In Tanzania, for example, a community health worker programme contacted more than 42,000 pregnant women over the course of 16 months. Of these, 75% had not yet attended antenatal care (including 40% of whom were in the first trimester) and were actively referred [101]. When the programme was formally evaluated via a cluster‐randomized trial, however, the proportion of women who reported fewer than four antenatal visits over the course of pregnancy did not differ between the intervention and standard‐of‐care arms (59.1% vs. 60.7%, RR: 0.97, 95% CI: 0.82 to 1.15). Similarly, the proportions of women who did not attend antenatal care in the first trimester also did not differ (69.7% vs. 70.3%, RR: 0.99, 95% CI: 0.87 to 1.13) [102].

Incentives may be promising. In a systematic review, Till et al. found that incentive‐based strategies may not increase the likelihood of antenatal care access, but pregnant women already attending antenatal care services were more likely to continue on a frequent basis [103]. In a small pilot trial in South Africa, Roussow and colleagues evaluated an intervention comprising the Thula Baba Box – modelled on the Finnish baby box and comprising maternal/newborn supplies – and monthly community health worker visits. The incentive was given conditional on early (i.e. within four weeks of the initial community health worker interaction) and continued (i.e. at least four antenatal care visits) engagement in antenatal care. Women randomized to the intervention arm appeared more likely to attend more than four antenatal care visits (adjusted odds ratio: 4.85, 95% CI: 0.84 to 27.88) and initiate antenatal care prior to five months’ gestation (adjusted odds ratio: 10.51, 95% CI: 1.80 to 61.83) [104].

Other strategies deserve consideration as well. Group antenatal care has gained considerable attention and is now recommended by the WHO [100]. Preliminary assessments of this approach have been encouraging, including increases in pregnancy‐related empowerment in some settings [105]. Larger trials of group antenatal care are nearing completion and should further solidify the evidence base for this strategy [106]. Similarly, SMS reminders may be effective for promoting antenatal care visits. In a systematic review and meta‐analysis, pregnant women who received text messaging were more likely to complete the four, focused antenatal care visits previously recommended by the WHO (OR: 2.74, 95% CI: 1.41 to 5.32) [107]. National programmes have begun to integrate such strategies into routine antenatal care [108]. Connections through internet‐based texting platforms (i.e. WhatsApp) could also replicate elements of patient group support and deserve further study.

### 3.6 Services for infants at high risk of HIV acquisition

When women initiate ART late in pregnancy, or fail to start at all, vertical HIV transmission rates of 30% and higher may be observed. The WHO considers the following groups at high risk of acquiring HIV: (1) infants born to women with established HIV infection who received less than four weeks of ART before delivery, (2) infants born to women with established HIV infection with viral load >1000 copies/mL in the four weeks before delivery, (3) infants born to women with incident HIV infection during pregnancy and breastfeeding, or (4) infants identified for the first time as HIV‐exposed during the postpartum period, with or without a negative HIV test prenatally [44]. Since maternal HIV antibodies are passively transmitted from mother to fetus and do not decay for months following birth, newborns should be screened via HIV nucleic acid tests.

For programmes with established capacity for early infant HIV diagnosis (EID) at age four to eight weeks and demonstrated links to paediatric HIV treatment services, the addition of HIV testing at birth should be considered. At present, infant HIV testing at birth has not been routinely incorporated in most public health settings, although there are exceptions. South Africa, for example has introduced universal birth testing for all HIV‐exposed infants because, in this setting of high HIV prevalence and limited maternal viral load testing, targeted birth HIV testing that is limited to “high risk” infants [109] may miss up to 20% to 25% of *in utero* HIV infections [110, 111]. While promising, the strategy of birth HIV testing could negatively affect routine early infant diagnosis (EID) testing at four to eight weeks for infants who initially test HIV‐negative [112]. For this reason, birth HIV testing programmes must be accompanied by careful evaluation of the impact on subsequent required EID testing and structured interventions may be needed to support follow‐up EID services after a negative HIV test at birth. Point‐of‐care assays for EID may facilitate implementation of birth HIV testing, although service delivery models may require further refinement [113, 114, 115]. Infants who test positive for HIV at birth require urgent linkages to HIV services, since early infant ART initiation has been shown to significantly reduce infant morbidity and mortality and neonatal ART initiation has been associated with more rapid viral suppression and restriction of viral reservoir size [116, 117, 118]. To achieve these benefits, however, the infrastructure to provide such services, including the correct antiretroviral formulations for newborns, must be in place.

All women newly diagnosed with HIV should start ART immediately, since maternal virological suppression is associated with improved maternal health and low rates of vertical HIV transmission. For infants at high risk for HIV acquisition, infant prophylaxis provides added antiretroviral coverage during the important window period between maternal ART initiation and maternal viral suppression. For breastfeeding infants born to HIV‐positive mothers on ART, the WHO currently recommends six weeks of infant prophylaxis with daily nevirapine, based on numerous clinical trials showing its efficacy [119, 120, 121, 122]. When infants are considered to be at high risk of mother‐to‐child HIV transmission, dual infant prophylaxis – with twice daily zidovudine and daily nevirapine – is recommended for the first six weeks of life, followed by an additional six weeks of dual prophylaxis or daily nevirapine for 12 week total duration in breastfeeding infants [44]. For infants at very high risk of HIV acquisition, a “presumptive treatment” regimen of three drugs (e.g. zidovudine + lamivudine + raltegravir) can be considered while awaiting results of a birth HIV test [123]. However, at least one diagnostic specimen must be collected prior to initiating any presumptive treatment, since these regimens could affect viral assay performance. Studies evaluating wide‐scale implementation of enhanced presumptive treatment strategies are not yet available.

## 4 DISCUSSION

The goal of eliminating mother‐to‐child HIV transmission has been an important driving force in global PMTCT programming and assessment [124]. To date, few countries have met the defined validation criteria for EMTCT – and none from high HIV burden settings [125]. The *Last Mile to EMTCT* is a data‐driven, iterative approach to help optimize PMTCT services, reduce new paediatric HIV infections, and move towards EMTCT milestones and validation criteria [2]. This operational guidance was developed from the recognition that, while countries worldwide are working to achieve EMTCT, few structured frameworks are available to assist national PMTCT programmes achieve these ambitious and important goals.

As a resource to support the *Last Mile to EMTCT*, we organized this review according to six intervention domains. Each of these intervention domains were further mapped to the different groups of pregnant and breastfeeding women to which – through the Spectrum PMTCT stacked bar analysis – new child HIV infections are attributed. Although these categories were designed to be distinct, we noted important overlap among identified interventions. For example engagement of male partners cut across the domains for HIV prevention services for women, timely access to HIV testing and programme retention and adherence support. Similarly, repeat HIV testing spanned two domains: HIV prevention services for women and timely access to HIV testing. Many domains were also repeated to address key programmatic gaps (Figure 2). This highlights the connected nature of many PMTCT interventions and, once implemented, emphasizes their potential synergy and broadened downstream impact.

Important, but outside the scope of this review, were strategies to link maternal and child health platforms with other units within and across health facilities. For example strategies that identify women with HIV, initiate them on ART, and help them to achieve viral suppression prior to conception could dramatically reduce the subsequent risk of horizontal and vertical HIV transmission. Strengthened links between sexual/reproductive health and HIV services, including integrated family planning models, could reduce the number of unintended pregnancies among women living with HIV.

We also recognize important cross‐cutting factors that contribute to the effectiveness of PMTCT interventions. Initiatives that reduce community‐level barriers (e.g. stigma and discrimination), for example could further increase demand for PMTCT services and positively influence uptake and retention. Given its prevalence in many settings, including those of high HIV burden, programmes that identify and address intimate partner violence may reduce its negative impact on health outcomes and empower women to seek care. Efforts to strengthen health systems and to fully deliver well‐implemented, quality services can also have important downstream benefits [126, 127, 128, 129, 130, 131]. Because such cross‐cutting approaches did not map to our *a priori* framework, however, they were not formally included in this review.

The overarching goal of this review was to summarize the current scientific evidence and practices in PMTCT, to further accelerate progress towards the goals of EMTCT. We view this as a resource for policy makers, programme managers and funding agencies as they seek to optimize PMTCT services. However, it is not intended to stand alone. Using the *Last Mile to EMTCT* framework [2], relevant strategies – addressing programme gaps identified by the stacked bar analysis – should be evaluated for suitability to the local context. The impracticality of some interventions may be immediately evident, especially at a population level, and these may be dismissed early. Interventions deemed feasible and appropriate should be prioritized according to their potential impact, cost and management (as well as other locally relevant criteria). Some interventions may work synergistically in combination with others, particularly in the support of adherence and retention. Instruments that assess the cost‐effectiveness of different approaches may help stakeholders to identify the optimal mix of interventions. New strategies must be considered alongside broader government priorities for maternal and child health, to better understand the overall impact of provided services.

## 5 CONCLUSIONS

This narrative review identified a broad evidence base of promising and proven interventions to address key gaps in PMTCT programmes and to broaden their reach and effectiveness. Used to support the *Last Mile to EMTCT* roadmap, this information can help country teams to systematically evaluate, plan and ultimately optimize PMTCT programmes at the national and sub‐national levels.

## COMPETING INTEREST

The authors declare no competing interest.

## AUTHORS’ CONTRIBUTIONS

BHC, DMN, SE and CL developed the initial concept. BHC conducted the review and prepared the manuscript. DMN, SE, LMM, FT, MM and CL contributed to the review and assisted in the interpretation of findings. All authors critically reviewed the manuscript for intellectual content. All have approved the final version for publication.
